# Study on the Control of Steam Front Mobility in High-Temperature and High-Salinity Conditions Using Polymer-Enhanced Foam

**DOI:** 10.3390/polym16172478

**Published:** 2024-08-30

**Authors:** Mingxuan Wu, Binfei Li, Liwei Ruan, Yongqiang Tang, Zhaomin Li

**Affiliations:** 1 Key Laboratory of Unconventional Oil & Gas Development, China University of Petroleum (East China), Ministry of Education, Qingdao 266580, China; 2 School of Petroleum Engineering, China University of Petroleum (East China), Qingdao 266580, China; 3 Sinopec Petroleum Exploration & Production Research Institute, Beijing 100083, China

**Keywords:** foam, steam front, enhanced oil recovery

## Abstract

This study investigated the enhancing effects of the temperature-resistant polymer Poly(ethylene-co-N-methylbutenoyl carboxylate-co-styrenesulfonate-co-pyrrolidone) (hereinafter referred to as Z364) on the performance of cocamidopropyl hydroxy sulfobetaine (CHSB) foam under high-temperature and high-salinity conditions. The potential of this enhanced foam system for mobility control during heavy oil thermal recovery processes was also evaluated. Through a series of experiments, including foam stability tests, surface tension measurements, rheological assessments, and parallel core flooding experiments, we systematically analyzed the interaction between the Z364 polymer and CHSB surfactant on foam performance. The results indicated that the addition of Z364 significantly improved the strength, thermal resistance, and salt tolerance of CHSB foam. Furthermore, the adsorption of CHSB on the polymer chains enhanced the salt resistance of the polymer itself, particularly demonstrating stronger blocking effects in high-permeability cores. The experimental findings showed that Z364 increased the viscosity of the liquid film, slowed down liquid drainage, and reduced gas diffusion, effectively extending the half-life of CHSB foam and improving its stability under high-temperature conditions. Additionally, in parallel core flooding experiments, the polymer-enhanced foam exhibited significant flow diversion effects in both high-permeability and low-permeability cores, effectively directing more fluid into low-permeability channels and improving fluid distribution in heterogeneous reservoirs. Overall, Z364 polymer-enhanced CHSB foam demonstrated superior mobility control during heavy oil thermal recovery, offering new technical insights for improving the development efficiency of high-temperature, high-salinity reservoirs.

## 1. Introduction

Heavy oil reservoirs are widely distributed around the world, with an estimated total reserve of 9–13 trillion tons [1]. One of the primary methods for enhancing heavy oil recovery is steam injection development [2,3]. To expand the steam sweep efficiency, a large amount of steam is typically required, which significantly increases energy consumption [4]. Previous studies have shown that adding non-condensable gases, such as nitrogen, during steam injection can effectively expand the steam sweep efficiency and accelerate the advancement of the steam front in heavy oil [5,6]. However, due to the high mobility ratio between gas and heavy oil, gas breakthrough often occurs rapidly. Foam, as a widely used technique for controlling gas flow in oil recovery processes, can mitigate the issue of steam channeling under the influence of non-condensable gases [7,8]. Injecting nitrogen foam at the steam front can effectively improve the utilization efficiency of the steam’s thermal energy [9].

The high-temperature environment and the presence of high-salinity water during steam injection impose greater demands on foam stability [10]. As more heavy oil reservoirs enter the steam injection development stage, zwitterionic surfactants, such as betaines, which exhibit excellent high-temperature foaming performance and salt tolerance, have garnered increased attention [11]. It has been reported that betaine foam can withstand salinity levels up to 2 × 10⁵ mg/L and temperatures as high as 150 °C [12,13,14].

Foam is a thermodynamically unstable structure [15]. In bulk foam, stability is primarily lost due to liquid drainage, gas diffusion, and film rupture [16,17,18]. After foam is generated, the fluid in the liquid films between the bubbles drains under the influence of gravity and the pressure difference described by the Prandtl boundary layer, leading to film thinning. The drainage process of the liquid film is a key factor affecting early foam stability [19,20]. As the liquid film thins, the diffusion of gas through the film from smaller bubbles to larger bubbles is enhanced. Gas diffusion is primarily influenced by the thickness and permeability of the surfactant adsorption layer and the middle layer of the liquid film [21,22]. The final rupture of the liquid film can be caused by excessive surface tension gradients resulting from the uneven distribution of surfactant molecules on the thin liquid film, or by flow-induced squeezing [23,24].

To improve foam stability, more suitable surfactants are selected based on the conditions in which the foam will be used. In addition to modifications in surfactant structure, methods such as blending with other surfactants, adding polymers to increase bulk viscosity, and incorporating nanoparticles have been proposed [25,26,27,28,29]. The main effects of these methods are to increase the viscosity of the liquid film, thereby slowing down drainage, to enhance the viscoelastic modulus of the film to improve foam’s resistance to disturbances, and to reduce gas diffusion.

Betaine surfactants exhibit good compatibility with other polymers and surfactants [30,31]. Although betaine surfactants demonstrate excellent thermal and salt tolerance, the addition of appropriate polymers can further enhance foam performance [32,33]. The polymer mainly improves foam stability by increasing liquid viscosity and forming surfactant–polymer complex structures with surfactant molecules [34]. There are various types of interactions between the polymer and surfactant, such as hydrophilic–hydrophobic interactions, electrostatic interactions, and spatial structure effects [33].

Cocamidopropyl hydroxysultaine (CHSB) is a high-temperature and high-salinity resistant surfactant, synthesized by bridging cocamidopropyl with betaine. This surfactant is characterized by its zwitterionic nature, containing ammonium ions, carboxyl groups, and sulfonic acid groups within its molecular structure. CHSB has been shown to exhibit excellent thermal and salt resistance in both experimental evaluations and field applications [34].

During the steam injection process in heavy oil reservoirs, a polymer designated as Z364 has been identified as an effective agent. This polymer’s backbone is composed of a long-chain saturated alkyl group −(CH_2_−CH_2_)_n_−, known for its high thermal stability. The polymer is synthesized from three monomeric structures: N-methylbutenoyl carboxylate, styrenesulfonate, and pyrrolidone. The N-methylbutenoyl carboxylate provides resistance to heat and CO_2_, while the styrenesulfonate group exhibits surface activity, aiding in the enhancement of the surfactant’s foaming properties. Pyrrolidone assists in adjusting the hydrophilicity of Z364, thereby improving the distribution of surfactant molecules at the gas–liquid interface.

In this study, a polymer-enhanced foam system based on the CHSB surfactant was investigated, with performance tests conducted on its salt tolerance and thermal stability. The viscosity and interfacial properties of the Z364 polymer and CHSB surfactant were evaluated to analyze the synergistic mechanisms between the polymer and surfactant. Additionally, the blocking efficiency of this foam system in different flow channels was examined. The results of this study provide valuable insights for controlling the mobility of high-temperature fluids during heavy oil recovery processes.

## 2. Materials and Methods

### 2.1. Material

**Figure 1:**
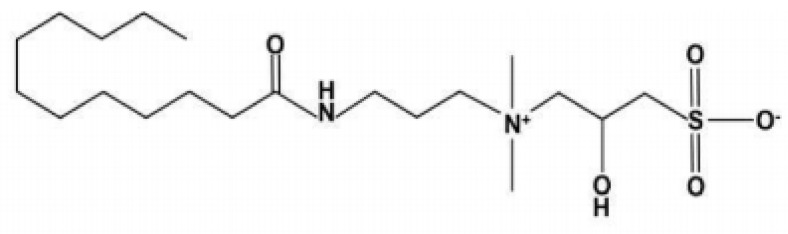
Molecular structure of CHSB.

The surfactant Cocamidopropyl Hydroxy Sulfobetaine (CHSB) (Figure 1) was purchased from Linyi Greensense Chemical Co., Ltd. (Linyi, China). The temperature-resistant polymer, designated as Z364, was provided by the Sinopec Research Institute of Petroleum Exploration and Development (Beijing, China). This polymer is suitable for various enhanced oil recovery (EOR) methods under high-temperature and high-salinity reservoir conditions. Z364 features a polymer backbone composed of highly thermally stable long-chain saturated alkyl groups −(CH_2_−CH_2_)n− and is a copolymer of N-methylbutenoyl carboxylate, styrenesulfonate, and pyrrolidone. This polymer exhibits excellent thermal stability, with the N-methylbutenoyl carboxylate monomer providing good heat and CO_2_ resistance, while the styrenesulfonate group imparts some surfactant properties that enhance foaming performance. The pyrrolidone component adjusts the hydrophilicity of Z364, thereby improving the distribution of surfactant molecules at the gas–liquid interface. The synthetic formation water used in the experiments was prepared with analytical grade NaCl and CaCl_2_ at a molar ratio of 6:1, to create different salinity levels. The nitrogen gas (99% purity) used in the experiments was supplied by the Qingdao Tianyuan Company (Qingdao, China).

### 2.2. Foamability and Foam Stability Assessment

Foam evaluation was conducted using the Waring Blender method. A total of 100 mL of solution, with a concentration range of 0.05–1%, was added to the stirring vessel. Prior to starting the mixer and during the mixing process, nitrogen gas was continuously injected into the chamber to ensure that the gas phase in the foam consisted of pure nitrogen. The instrument was set to operate at 8000 revolutions per minute, with a mixing duration of 180 s. The generated foam was then transferred to a graduated cylinder, where the foam volume (V_f_) and the volume of liquid drained (V_l_) were recorded over time. The foam drainage half-life (t_1/2_) was defined as the time required for 50% of the liquid to drain from the foam. This experimental procedure can be conducted in a heated chamber with a viewing window, depending on the experimental conditions.

### 2.3. Surface Property Measurements

Surface tension was measured using an interfacial tensiometer (Tracker-H, Teclis, Civrieux-d’Azergues, Lyon, France; pressure range: 0.1–20.0 MPa, temperature range: 0–200 °C). The surface tension of the surfactant solution was determined using the pendant drop method. Under program control, a droplet of surfactant solution with a volume of 6–8 microliters was formed at the tip of a needle, and a camera captured the changes in the droplet’s shape and volume over time. The software automatically calculated the dynamic surface tension and equilibrium surface tension.

### 2.4. Viscosity Measurements

The viscosity of the solution was measured using a modular rheometer (Anton Paar MCR302, Anton Paar GmbH, Graz, Austria). A cone–plate measurement system was employed, with a measurement gap of 0.102 mm and a plate diameter of 105 mm. The shear rate was set at 10 s^−1^, and all experiments were conducted at room temperature. The viscosity of each sample was tested 3 to 5 times, and the final result was obtained by averaging these measurements.

### 2.5. Core Plugging Experiment

**Figure 2:**
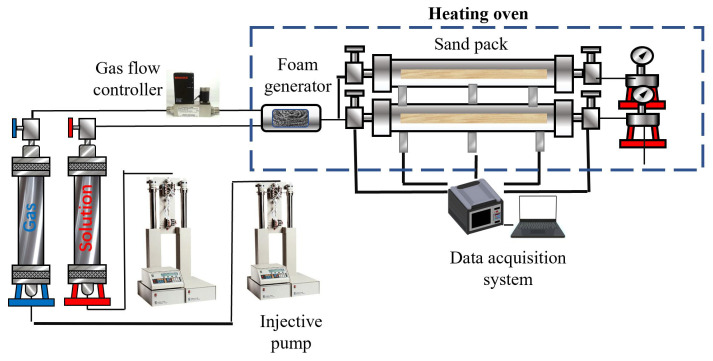
Flowchart of the core experiment.

First, 40–70 mesh quartz sand and 120 mesh quartz sand were packed into the sand-packed tube in ratios of 4:1 and 2:1, respectively. The permeability of the different cores was then measured using brine [35]. An initial injection of 1 pore volume (PV) of surfactant solution was conducted to ensure adsorption equilibrium on the core surface. The temperature of the core holder was then adjusted to 90 °C, and the back pressure of the equipment was set. Foam was injected into the core, followed by the injection of 90 °C hot water after a certain PV of foam had been introduced. During the experiment, the pressure values at the inlet, middle, and outlet of the core holder were recorded, along with the fluid production at the outlet. The experimental procedure is illustrated in Figure 2.

## 3. Results and Discussion

### 3.1. Salt Tolerance of Betaine Foam

**Figure 3:**
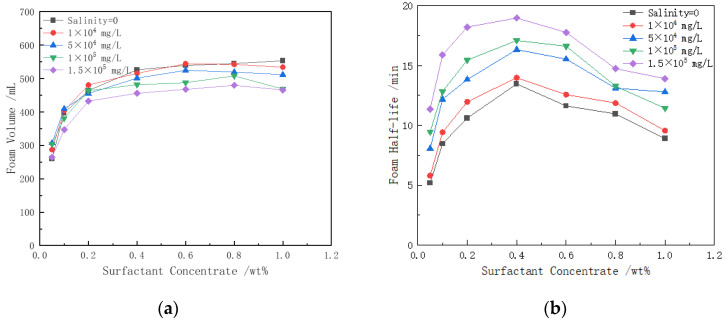
The effects of salt solutions with different concentrations on foam characteristics: (**a**) foam volume; (**b**) foam half-life. (50 °C).

To investigate the relationship between the foaming ability and foam stability of the surfactant under different salinity conditions and varying surfactant concentrations, we conducted a series of experiments. The experimental results are shown in Figure 3. The foam volume increases with the increase in surfactant concentration and the decrease in salinity. The half-life of the foam drainage also increases as both the surfactant concentration and salinity increase. However, when the surfactant concentration exceeds 0.4%, it negatively impacts the foam stability.

As shown in Figure 3a, in a salt solution of a given concentration, when the concentration of the surfactant CHSB is 0.05 wt%, the foam volume ranges between 260 and 307 mL, indicating a relatively small foam volume. This is due to the low concentration of CHSB, which results in the insufficient adsorption of surfactant molecules at the gas–liquid interface, preventing the formation of a compact monolayer and consequently leading to poor foam volume and stability. As the surfactant concentration increases to 0.4 wt%, the adsorption of surfactant molecules at the gas–liquid interface becomes more dense, thereby enhancing the foaming ability and foam stability of the solution. However, when the CHSB concentration is further increased, a large number of micelles form in the solution. The electrostatic repulsion between the ionic groups within the micelles reduces the Marangoni effect, which is essential for maintaining foam stability, leading to a decrease in foam stability [36]. Although this phenomenon does not significantly affect foam generation, it negatively impacts foam maintenance [37]. The experimental results indicate that the foam stability is optimal when the CHSB concentration is around 0.4 wt%.

Salt ions in high-salinity water have a significant impact on the properties of foam. In surfactant solutions of a given concentration, as the concentration of salt ions increases, the foam volume tends to decrease. For example, in a 1 wt% CHSB solution, when the salinity increases from 0 to 1.5 × 10⁵ mg/L, the foam volume decreases by up to 21.7%. However, in low-concentration CHSB solutions, the effect of salt ions on foam volume is less pronounced. Unlike the generally negative effects that salt ions have on the foam stability of anionic or cationic surfactants, salt ions have a beneficial effect on the stability of CHSB foam [37]. As shown in Figure 3b, as salinity increases, foam stability correspondingly improves. For instance, in a 0.4 wt% surfactant solution, when the salinity increases from 0 to 1.5 × 10⁵ mg/L, the foam half-life increases from 13.5 min to 18.9 min. Notably, at a CHSB concentration of 0.05 wt%, the foam half-life increases by a factor of 2.2, showing the most significant effect.

After CHSB foam is formed, the first phenomenon that occurs is the drainage of the liquid film. The addition of salt ions can screen the electrostatic repulsion within the double layer of surfactant molecules, allowing CHSB surfactant molecules to form a denser monolayer or multilayer at the gas–liquid interface. This increases the thickness of the liquid film and prolongs the drainage time [38]. While the liquid film is draining, gas also diffuses through the film between bubbles of different sizes, accompanied by foam coalescence triggered by film rupture. The thickening of the liquid film and the reduction of drainage slow down the gas diffusion across the film, thereby delaying the thinning and rupture of the film. Additionally, an increase in salt ion concentration allows surfactant molecules to more quickly approach and adsorb onto the gas–liquid interface, which enhances the Marangoni effect and helps maintain the stability of the liquid film. This series of effects delays foam coalescence, thereby improving the overall stability of the foam [39].

### 3.2. The Effect of Polymer on Foam Stability

**Figure 4:**
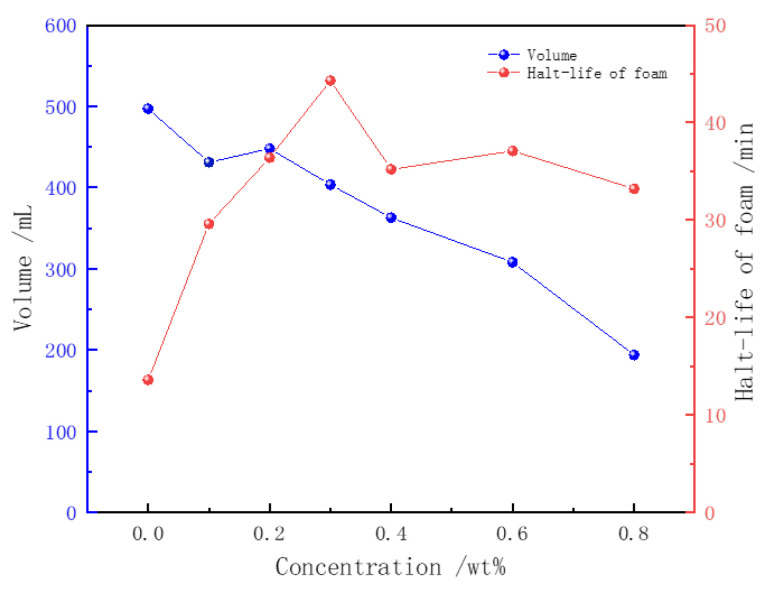
The effect of polymer concentration on foam characteristics (CHSB concentration: 0.4%, salinity: 0, temperature: 50 °C).

The impact of the Z364 polymer on betaine foam was evaluated using the Waring Blender method. The foam volume and drainage half-life are shown in Figure 4. With a consistent concentration of CHSB, the addition of the polymer significantly increased the foam’s drainage half-life, while simultaneously reducing the foam volume. At a polymer concentration of 0.3%, the drainage half-life of the foam increased dramatically from 13.6 min to 44.3 min, representing a 325% increase. However, with further increases in polymer concentration, the effect on extending the foam’s half-life became limited.

The incorporation of the Z364 polymer enhanced the viscosity of the foam liquid film, thereby slowing down the liquid drainage rate and the diffusion of gas through the film. More importantly, CHSB surfactant molecules formed a complex with Z364 molecules. The carboxylate groups (-COO^−^), sulfonate groups (-SO_3_^−^), and carbonyl groups (C=O) in the polymer can form hydrogen bonds with the hydroxyl, amide, or other polar groups in CHSB. Additionally, the negatively charged carboxylate and sulfonate groups can form ionic bonds with the positively charged regions of CHSB. The interaction between the long-chain saturated alkyl groups in the polymer and the hydrophobic long chains of CHSB enhances molecular hydrophobicity, thereby increasing the distribution density at the gas–liquid interface. This series of synergistic interactions helps to form a stable bilayer structure of polymer–surfactant complexes at the gas–liquid interface, thereby improving foam stability [33].

The addition of the polymer had a negative impact on the foaming ability of CHSB, and the foam volume decreased rapidly with increasing polymer concentration. This is because the Z364 polymer competes with CHSB molecules for adsorption at the gas–liquid interface, reducing the effective concentration of CHSB molecules. Furthermore, the increase in polymer concentration raises the viscosity of the solution, which in turn increases the energy required to form the gas–liquid interface [40]. The higher viscosity of the solution also slows down the transfer of surfactant molecules to the gas–liquid interface, thereby weakening the surfactant’s ability to reduce surface tension. The higher the concentration of the Z364 polymer, the stronger its inhibitory effect on the foaming ability of the surfactant.

### 3.3. Thermal Stability of Foam

**Figure 5:**
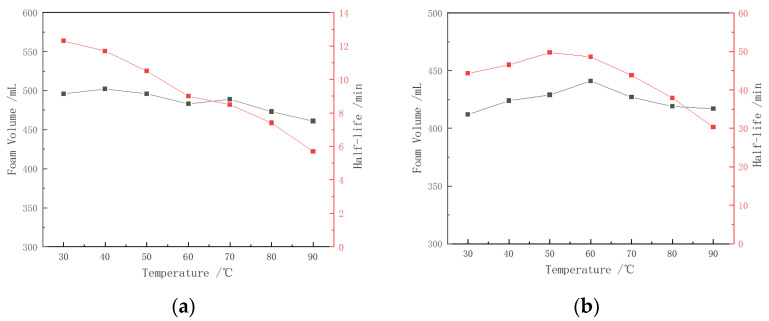
Thermal stability of pure surfactant foam and polymer-enhanced foam: (**a**) 0.4% CHSB, (**b**) 0.4% CHSB + 0.3% Z364. (Salinity: 0).

The effects of temperature on the two types of foam are shown in Figure 5. Although the CHSB surfactant has relatively good thermal stability, increasing temperatures still negatively impact foam stability. As shown in Figure 5a, within the temperature range of up to 50 °C, the increase in temperature reduces the viscosity of the solution and enhances the migration speed of surfactant molecules at the gas–liquid interface, thereby lowering the surface tension and increasing foam volume. As a result, the solution exhibits improved foaming ability. However, as the temperature continues to rise, more surfactant molecules form micelles [41], leading to a decrease in the number of molecules adsorbed at the gas–liquid interface. This reduces the surface tension gradient, weakening the strength and self-repair ability of the liquid film, causing the foam volume to begin declining [20,24]. The rise in temperature not only destabilizes the liquid film and increases the probability of film rupture but also accelerates the thermal motion of gas molecules, increasing the coalescence rate between bubbles of different sizes [33,41]. The drainage half-life of CHSB foam decreases significantly, with the foam half-life at 90 °C being reduced by 54.7% compared to that at 30 °C.

The addition of the polymer significantly enhances the thermal stability of CHSB foam. The Z364 polymer not only maintains good physical and chemical properties at high temperatures due to its stable backbone structure, but its amide and sulfonate functional groups can also form hydrogen bonds with water molecules, thereby improving the water retention of the foam liquid film. Through the synergistic interaction with the CHSB surfactant, the viscosity of the CHSB surfactant solution was increased, and the stability of surfactant molecule adsorption at the gas–liquid interface was enhanced. The drainage effect of the liquid film was suppressed, and the film strength was improved. As a result of these combined effects, the stability and thermal resistance of the foam were enhanced [12,42]. This, in turn, improves the stability and thermal resistance of the foam. Below 60 °C, the increase in temperature does not weaken the adsorption of the surfactant and polymer at the gas–liquid interface; rather, it prolongs the half-life of the foam and increases foam volume. However, as the temperature continues to rise, the strength of the liquid film in the polymer–surfactant mixed foam decreases, and the probability of rupture increases, resulting in a reduction in the foam’s drainage half-life as the temperature increases [43].

### 3.4. Viscosity Analysis

**Figure 6:**
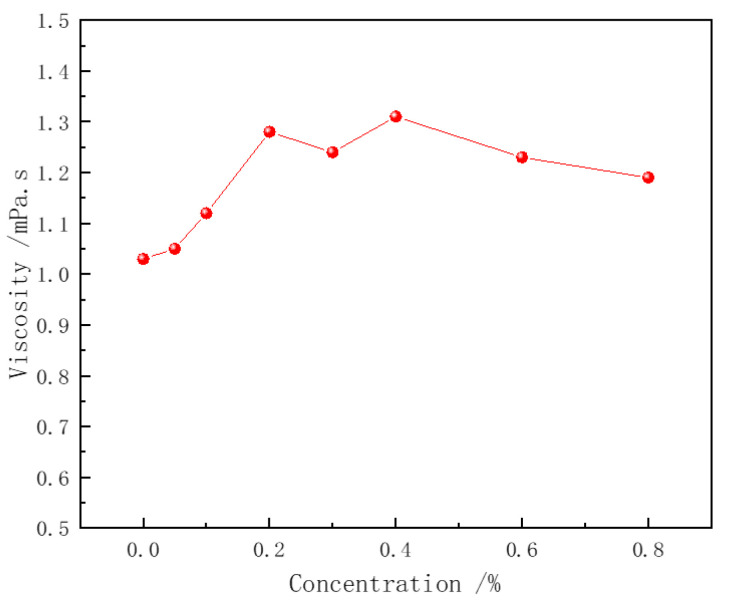
Viscosity of CHSB solution. (Polymer concentration: 0%, Salinity: 0%, 25 °C).

**Figure 7:**
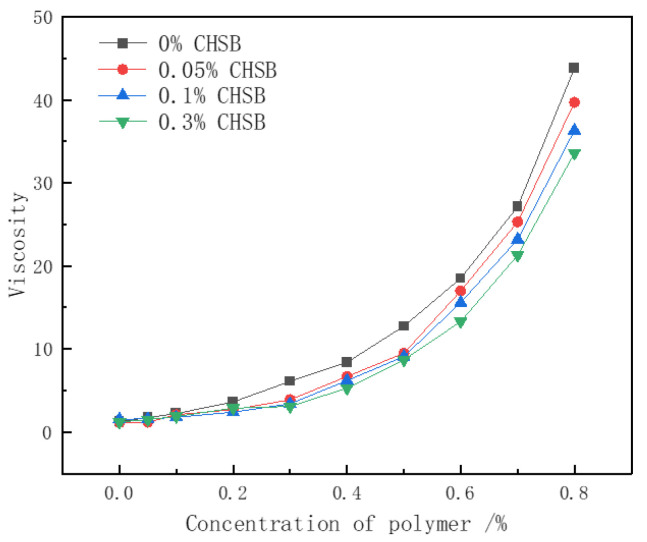
Relationship between Z364 polymer viscosity and concentration. (Salinity: 0%, 25 °C).

The thickening ability of the polymer is one of the key mechanisms by which it enhances the stability of surfactant foam. In the pure CHSB solution, a minimal effect on the solution’s viscosity was observed with the increase in surfactant concentration (as shown in Figure 6). However, Figure 7 shows that the viscosity of the polymer solution increases exponentially with the polymer concentration, but decreases when a certain concentration of CHSB is added. As the polymer concentration increases, the polymer chains become more entangled, forming a network structure. The number of entanglement points grows non-linearly with the increase in concentration, leading to an exponential rise in the solution’s viscosity. This occurs because CHSB surfactant molecules electrostatically adsorb onto the polymer chains, weakening the electrostatic repulsion and van der Waals forces between the polymer chains [12,44]. Simultaneously, the hydrophobic tail groups of CHSB interact with the hydrophobic segments of the Z364 polymer chains, further shielding the interactions between the polymer chains. This effect is particularly pronounced under higher concentrations of CHSB [45].

**Figure 8:**
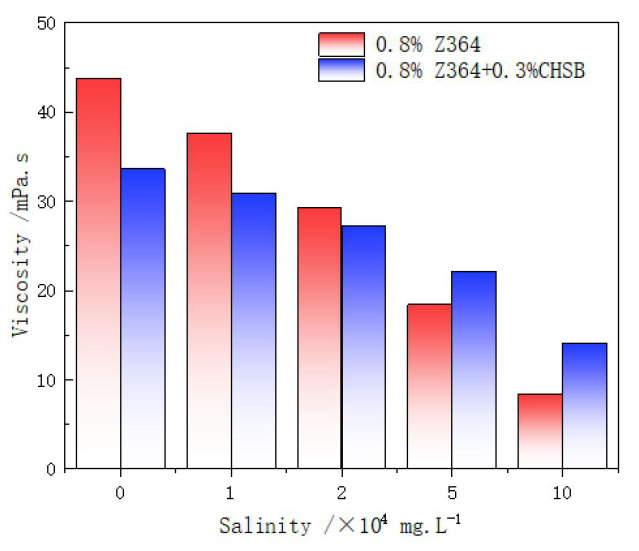
Viscosity analysis of the solution under different salinities (25 °C).

The viscosity of the polymer in synthetic formation water is shown in Figure 8. As the salinity increases, the thickening effect of the polymer significantly decreases. The salt ions in the synthetic formation water shield the negative charges on the polymer, reducing electrostatic repulsion and causing the polymer chains to contract [22,46]. This effect becomes more pronounced with increasing salinity.

However, the addition of the CHSB surfactant improved the salt tolerance of the polymer in high-salinity water. The viscosity of the polymer solution containing CHSB decreases less with increasing salinity compared to the pure Z364 solution. This is closely related to the chemical structure of the polymer and the surfactant. The cationic head group of CHSB forms strong electrostatic attractions with the anionic functional groups (-COO^−^ and -SO_3_^−^) on the Z364 polymer chains. This interaction effectively shields the polymer chains from the effects of the high external salt concentration, reducing the interference of salt ions. Additionally, the hydrophobic tail of CHSB interacts with the hydrophobic segments of the Z364 polymer chains, forming hydrophobic microdomains. This self-assembled structure is more stable in high-salinity environments, thereby increasing the solution’s viscosity. The interaction between the hydrophobic tails also helps to form a stable polymer network structure, enhancing the viscosity and structural strength of the solution [23,45].

### 3.5. Analysis of Surfactant Surface Tension

**Figure 9:**
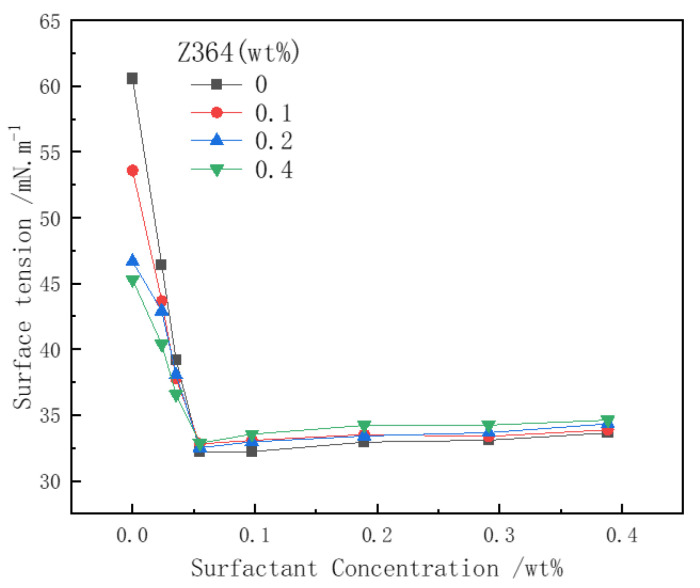
The effect of polymer on surfactant surface tension. (Salinity: 0%, 25 °C, 0.1 MPa).

Surface tension is one of the key factors influencing foam generation and stability. It is well known that the lower the surface tension, the less energy is required to form the foam liquid film. The addition of polymers can affect the adsorption state of surfactant molecules at the gas–water interface, thereby influencing foam generation and stability [11,47]. Figure 9 illustrates the relationship between the surface tension of the surfactant solution with nitrogen and the changes in surfactant and polymer concentrations. As the concentration of CHSB increases, the surface tension of the solution decreases sharply, with the surface tension inflection point occurring at a surfactant concentration of approximately 0.05 wt%. For pure Z364 polymer solutions, the surface tension also decreases with increasing concentration, indicating that the polymer itself possesses some surface activity [12,22]. In the mixed solution, due to electrostatic interactions and hydrophobic chains, the polymer reduces the adsorption of surfactant at the gas–liquid interface, thereby increasing the surface tension [48].

**Figure 10:**
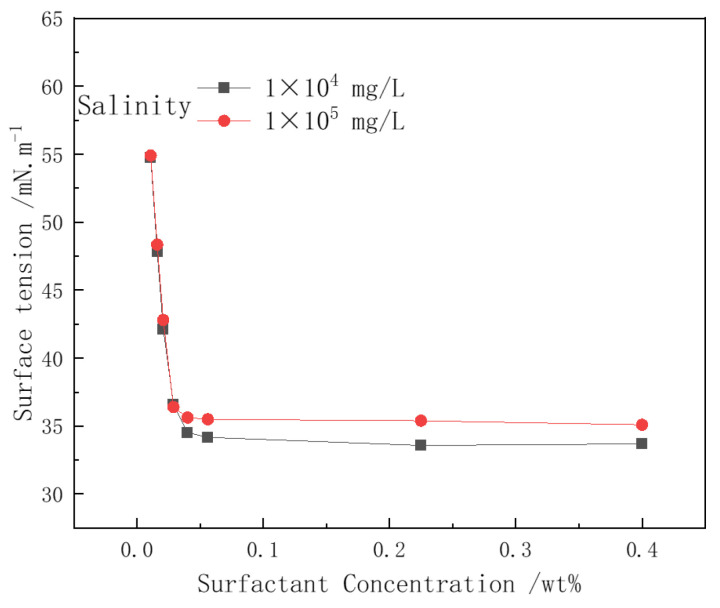
The effect of salinity on surfactant surface tension. (Polymer concentration: 0%, 25 °C, 0.1 MPa).

The surface tension of CHSB solutions with different concentrations was measured under salinity conditions of 1 × 10^4^ mg/L and 1 × 10^5^ mg/L (Figure 10). The hydrophilic groups of CHSB include an anionic sulfonate group and a cationic quaternary ammonium group. In salt solutions, there is electrostatic attraction between the oppositely charged ions of the surfactant molecules and electrostatic repulsion between ions of the same charge. The salt ions’ electrostatic shielding effect on the surfactant is weakened by the electrostatic attraction between counter-ions [12,47,48]. When the concentration of CHSB exceeds the CMC (Critical Micelle Concentration), the salt ions in the high-salinity water help improve the adsorption of surfactant molecules at the gas–liquid interface, slightly reducing the surface tension. At a salinity of 1 × 10^5^ mg/L, the amount of surfactant molecules adsorbed at the gas–liquid interface does not significantly decrease, and the surface tension only slightly increases [24,35,40]. It is important to note that the addition of salt ions to the solution lowers the CMC value of CHSB, which also implies that the same concentration of CHSB solution may have a higher CMC multiple. This could have a certain impact on the stability of the foam liquid film.

### 3.6. Foam Flow Characteristics in Porous Media

#### 3.6.1. Pressure Analysis

**Figure 11:**
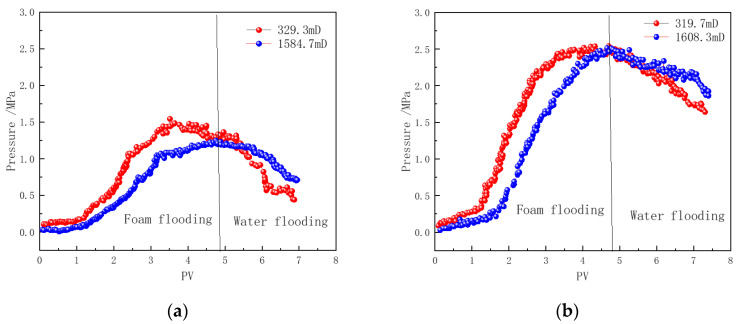
Pressure profiles of foam flow in core flooding: (**a**) CHSB foam, (**b**) polymer-enhanced CHSB foam.

In heavy oil reservoirs, in addition to the inherent heterogeneity of the reservoir itself, the high-permeability channels formed by the displacement fluid in the heavy oil further exacerbate the heterogeneity of the flow channels during subsequent displacement processes [49,50]. To investigate the selective plugging effect of polymer-enhanced foam on high-permeability and low-permeability channels in the reservoir, parallel core flooding experiments were conducted, with the experimental parameters shown in Table 1. The gas volume fraction (foam quality) in the foam is 80%. Figure 11 presents the pressure difference curves between the middle and outlet positions of the cores. The results indicate significant differences in the flow process and stability of the foam in high-permeability and low-permeability cores.

During the injection of CHSB foam, the foam preferentially enters the high-permeability core. In the pores of the high-permeability core, due to the fast gas–liquid flow rate and strong shear effects, the foam undergoes frequent “breakdown-regeneration” cycles in the high-permeability formation [51]. As a result, the pressure drop is greater in the front section of the high-permeability core, while the pressure at the middle measurement point is lower. In contrast, the pressure drop in the front half of the low-permeability core is smaller, and the Jamin effect caused by the “breakdown-regeneration” process of the foam in the core is significantly weaker than in the high-permeability core [10,50,51]. This indicates that the injected fluid is effectively diverted into the low-permeability core.

As the number of injected pore volumes (PVs) increases, stable foam flow is established in both cores, and the pressure characteristics in the middle sections of the cores gradually become consistent. Although the intensity of the Jamin effect, caused by the generation and collapse of foam, differs between the high-permeability and low-permeability cores, the pressure distribution patterns in the high-permeability and low-permeability cores become similar during the stable foam flow stage [52,53].

During the hot water injection stage, nitrogen injection at the core inlet is stopped. Due to the lack of nitrogen disturbance and replenishment, the stability of the foam in cores with different permeabilities has a more significant impact on the pressure curves. The pressure in the middle section of the low-permeability core decreases rapidly, while the pressure in the middle section of the high-permeability core remains at a relatively high level. The high-permeability core forms stronger foam strength during the foam flow stage, and the foam stability is better. Even though the hot water flush reduces foam strength in the front section of the core, the foam in the latter half still maintains a substantial local pressure difference. In contrast, the pressure in the middle section of the low-permeability core drops more quickly, indicating that the foam stability within the core is compromised, and more injected fluid is diverted into the low-permeability core, further accelerating foam collapse [18,53]. Throughout the foam injection and subsequent fluid flow processes, CHSB foam effectively directs more fluid into the low-permeability core.

Compared to pure CHSB foam, the foam enhanced with the Z364 polymer exhibits significantly higher flow resistance in both high-permeability and low-permeability cores. Although the flow patterns of the two types of foam in the parallel cores are similar, both achieving effective fluid diversion with more fluid entering the low-permeability core, the addition of the polymer not only strengthens the foam but also improves its thermal stability [12,22,24]. Consequently, during the subsequent hot water flooding process, the rate of pressure decline in both cores is slower than that observed in the first set of experiments.

By comparing the pressure data of the two cores during the water flooding stage in Figure 11b, it is evident that the addition of the polymer significantly improves the foam stability in the low-permeability core [8,54]. In the application of steam-assisted heavy oil recovery, this enhanced plugging performance in low-permeability channels facilitates more effective displacement of previously untouched heavy oil by the subsequent injected fluid.

#### 3.6.2. Flow Rate Analysis

**Figure 12:**
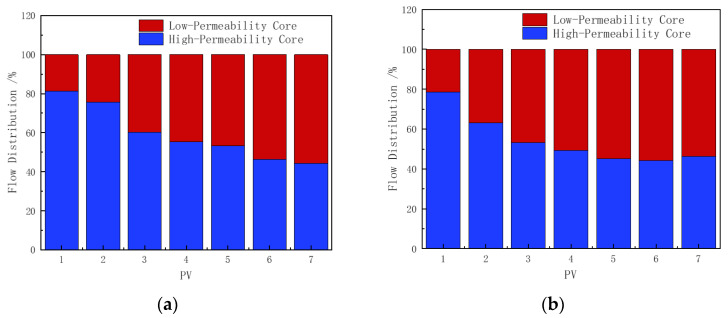
Flow distribution in parallel cores: (**a**) CHSB foam, (**b**) polymer-enhanced CHSB foam.

Both types of foam improved the flow distribution between the high-permeability and low-permeability cores in the parallel core setup, as shown in Figure 12. The polymer-enhanced CHSB foam exhibited stronger plugging performance in the high-permeability core. During the injection of 0–1 PV, the foam blocking effect in the core gradually established, with the regulatory effect of the polymer-enhanced foam being slightly superior to that of the CHSB foam. During the 1–3 PV injection period, the flow rate in the low-permeability core in Figure 12b increased by 12.4% and 7.2% compared to the low-permeability core in Figure 12a [50,55]. Combined with the corresponding pressure curves, it can be inferred that as the polymer-enhanced foam entered the high-permeability core, the Jamin effect in the low-permeability core was weaker.

During the 4–5 PV foam injection period, foam in both the high-permeability and low-permeability cores reached a stable flow state [35]. The flow rate of the polymer-enhanced foam in the low-permeability core was 6.2–8% higher. During the subsequent injection of 90 °C hot water, the poor stability of the pure CHSB foam caused the foam in the low-permeability core to destabilize more quickly under the flush of surfactant-free hot water, leading to a further increase in the flow rate through the low-permeability core [56]. However, this advantage turns into a disadvantage as the foam in the high-permeability core begins to collapse.

## 4. Conclusions

(1) The presence of salt ions has a significant impact on the formation and stability of CHSB foam. While high-salinity conditions may inhibit the foaming ability of the surfactant, they can also enhance foam stability. The formation of a stable self-assembled structure between CHSB and the Z364 polymer strengthens the polymer’s salt tolerance, increases the solution’s viscosity, and enables the foam to maintain good stability and flow control capabilities in high-salinity environments.

(2) The impact of temperature on CHSB foam is primarily reflected in its stability and foaming ability. Below 50 °C, an increase in temperature promotes foam generation and increases foam volume. However, as the temperature rises further, the micellization effect of the surfactant molecules weakens foam stability. In contrast, Z364-enhanced foam exhibits better tolerance and stability under high-temperature conditions, which slows down foam decay.

(3) In the parallel core flooding experiments, the flow resistance of the polymer-enhanced CHSB foam was significantly increased in both high-permeability and low-permeability cores. The addition of the polymer, particularly in the low-permeability core, effectively guided more fluid into the low-permeability channels by enhancing the solution’s viscosity and the adsorption ability of the surfactant molecules, thereby improving fluid distribution in heterogeneous reservoirs.

## Figures and Tables

**Table 1:** Foam core flooding experiment data.

No.	Permeability/mD	Porosity/%	Solution	Foam Quality
1-1	329.3	26.58	0.4% CHSB	80%
1-2	1584.7	28.54	0.4% CHSB	80%
2-1	319.7	27.46	0.4% CHSB + 0.3% Z364	80%
2-2	1608.3	29.91	0.4% CHSB + 0.3% Z364	80%

## Data Availability

The raw data supporting the conclusions of this article will be made available by the authors on request.
